# Tunneling and Suture of Thoracic Epidural Catheters Decrease the Incidence of Catheter Dislodgement

**DOI:** 10.1155/2014/610635

**Published:** 2014-07-21

**Authors:** Timur Sellmann, Victoria Bierfischer, Andrea Schmitz, Martin Weiss, Stefanie Rabenalt, Colin MacKenzie, Peter Kienbaum

**Affiliations:** ^1^Klinik für Anästhesiologie, Universitätsklinikum Düsseldorf, Moorenstraße 5, 40225 Düsseldorf, Germany; ^2^Institut für Medizinische Mikrobiologie und Krankenhaushygiene, Universitätsklinikum Düsseldorf, Moorenstraße 5, 40225 Düsseldorf, Germany

## Abstract

*Background.* Dislocation of epidural catheters (EC) is associated with early termination of regional analgesia and rare complications like epidural bleeding. We tested the hypothesis that maximum effort in fixation by tunneling and suture decreases the incidence of catheter dislocation. * Methods.* Patients scheduled for major surgery (*n* = 121) were prospectively randomized in 2 groups. Thoracic EC were subcutaneously tunneled and sutured (tunneled) or fixed with adhesive tape (taped). The difference of EC length at skin surface level immediately after insertion and before removal was determined and the absolute values were averaged. Postoperative pain was evaluated by numeric rating scale twice daily and EC tips were screened microbiologically after removal. * Results.* Both groups did not differ with respect to treatment duration (tunneled: 109 hours ±46, taped: 97 ± 37) and postoperative pain scores. Tunneling significantly reduced average extent (tunneled: 3 mm ±7, taped: 10 ± 18) and incidence of clinically relevant EC dislocation (>20 mm, tunneled: 1/60, taped: 9/61). Bacterial contamination showed a tendency to be lower in patients with tunneled catheters (8/59, taped: 14/54, *P* = 0.08). * Conclusion.* Thorough fixation of EC by tunneling and suturing decreases the incidence and extent of dislocation and potentially even that of bacterial contamination.

## 1. Introduction

Dislocation of epidural catheters (EC) may cause early termination of postoperative regional analgesia. Moreover, accidental removal shortly after anticoagulant administration, such as prophylaxis of deep vein thrombosis, may increase the risk of epidural hematoma and neurologic complications [1, 2]. Finally, it is speculated that catheter movement within the skin may potentially contribute to bacterial contamination possibly linked to catheter-related infective complications with colonization rates as high as 12% [3].

At our institution, EC had been traditionally attached to the skin using adhesive tape (taped). Regarding institutional data, dislocation occurred in up to 30 percent of our patients during the first postoperative days, which is within previously reported limits [4]. Accordingly, we tested the hypothesis that maximum effort to secure EC by subcutaneous tunneling and suture decreases the incidence of dislocation and the extent of movement. Postoperative analgesia during EC treatment as quantified by numeric rating scale and bacterial contamination was defined as secondary study endpoints.

## 2. Material and Methods

### 2.1. Ethics

Ethical approval for this study (Ethical Committee ID number 3433) was provided by the Ethical Committee of the Medical Faculty of the University of Düsseldorf, Düsseldorf (Chairperson Professor Dr. H.-G. Lenhard), on July 28, 2010. Additionally the study was registered at clinicialtrials.gov (http://www.clinicaltrials.gov/, NCT01402778).

**Figure 1:**
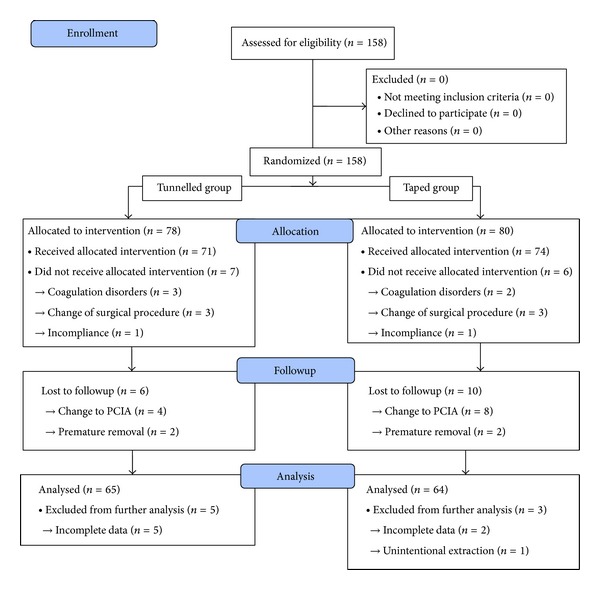
Flow chart according to CONSORT guidelines. PCIA patient controlled intravenous anesthesia.

After informed consent, 158 patients older than 18 years and scheduled for major abdominal or thoracic surgery under combined general and thoracic epidural anesthesia were assessed for eligibility (Figure 1). Patients were allocated to treatment groups by means of randomization (block formation with 10 patients each).

### 2.2. Treatment Groups

Thoracic EC were inserted preoperatively before induction of anesthesia using the “loss of resistance technique” under sterile conditions using gloves, surgical caps, gown, and facial mask.

**Figure 2:**
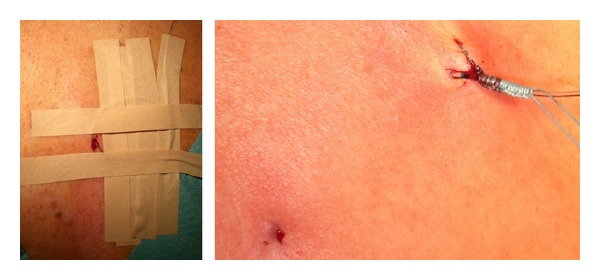
Different fixation techniques. Fixation by taping (left) and tunneling and suturing (right). For further information please refer to the text.

Patients were placed in sitting position and their backs were prepped with a propanol-based solution (Kodan tincture forte, Schuelke & Mayr GmbH, Norderstedt, Germany) for 2 minutes and covered with a fenestrated self-adhesive drape [5]. A skin wheal was induced using lidocaine 1%, followed by the insertion of a 17-gauge Tuohy needle. After loss of resistance to saline, an EC (Perifix Catheter, B. Braun, Germany) was inserted 3–5 cm into the epidural space and connected to a Perifix bacterial filter (0.2 *μ*m; B. Braun, Germany). EC were either fixated by steri-strips (Steri-Strip, 3M, St. Paul, MN, USA) or subcutaneously tunneled (>2 cm) using a 16-gauge i.v. line as a control structure followed by suturing to the skin using a synthetic, monofilament, nonabsorbable polyester suture. Steri-strips were taped leaving the puncture site uncovered. Thus, the position of the catheter could be assessed without movement likely induced by removal of the sterile tapes. Fixation techniques are presented in Figure 2. Afterwards, all EC were covered at the puncture site with a sterile tape (Tegaderm 3M, St. Paul, MN, USA). The distance between epidural tip and skin surface was recorded in each patient.

Postoperative analgesia was accomplished using epidural ropivacaine 0.2% (4–10 mL/h, depending on NRS score). Comedication consisted of intravenous metamizole (1 g every 6 h). In case of intolerance intravenous paracetamol (1 g every 6 h) was given. Intravenous piritramide (7.5 mg) was allowed as rescue medication.

### 2.3. Postoperative Followup

A physician of the Acute Pain Service (APS) daily visited all patients twice until 24 hours after catheter removal. No systematic change of drapes was undertaken. There was no specific nurse protocol for EC maintenance.

Pain intensity (numeric rating scale, NRS) at rest and during movement, use of analgesic adjunct, systemic antibiotic medications, and signs of catheter-related local complications were assessed during follow-up visits. Duration of treatment and time point of sterile catheter removal were determined by an anesthesiologist not involved in the study. The catheter tip was transferred to a polypropylene screw-cap tube with internal conical shape filled with 1 mL of liquid Amies medium (Copan Innovation, Brescia, Italy) for microbiological evaluation.

### 2.4. Study Endpoints

#### 2.4.1. Epidural Catheter Dislocation

The distance between catheter tip and the skin was recorded a second time at removal and compared to the preoperative value directly after catheter insertion. Absolute values for catheter length were determined in millimeters using a ruler. Data collection and catheter removal were performed by an anesthesiologist who was blinded to the initial value at catheter insertion. According to previous definitions [4, 6] and the type of multiorifice catheters used, we considered dislocation to be clinical relevant when in- or outward movement greater than 20 mm occurred.

#### 2.4.2. Quality of Postoperative Analgesia

The extent of postoperative analgesia was recorded after interviewing the patients using NRS at rest and during movement. After catheter removal, overall subjective contentment with the procedure was assessed retrospectively, using notes from 1 (excellent) to 5 (insufficient).

#### 2.4.3. Clinical Signs of Infection

Clinical signs of site inflammation followed the classification recommended by the German Society of Anesthesiologists and were defined as mild (two or more of the following: redness, swelling, pressure pain at catheter insertion, or tunneling site), moderate (two or more of the following: rise of C-reactive protein, pus secretion from puncture site, leukocytosis, fever, or necessity for antibiotics after exclusion of other causes), or severe (need for surgical intervention) [6–10].

#### 2.4.4. Bacterial Contamination

The catheter tip was cut into roughly 5 mm pieces that were incubated in thioglycolate bouillon for 48 hours. The cultures were assessed at 24 and 48 hours and if growth was detected a Gram stain was performed and 10 *μ*L aliquot of the bouillon was plated onto MaConkey, blood, and chocolate agar, respectively. If yeasts were seen on Gram staining Sabouraud agar was inoculated. The agar plates were incubated for 24 hours and microbiological methods were used to identify the bacteria. Bacteria were then tested for antibiotic sensitivity.

### 2.5. Statistical Analysis

Sample size calculation: assuming an incidence of clinical relevant EC migration (>20 mm) at our institution in 27% of patients (±10%) with traditional EC fixation a 15% difference (incidence greater than 31% or lower than 23%) can be determined by inclusion of 60 patients per group (*α* < 0.05, 1 − *β* < 0.2).

Data are expressed as mean (SD) except ordinal data (median, interquartile range). Statistical analysis was performed using Fisher's exact test, student's *t*-test, Mann-Whitney test, or Friedman's test when appropriate. *P* values less than 0.05 were considered to be statistically significant.

## 3. Results 

Sixteen patients (10 taped and 6 tunneled) were lost to followup, 7 had incomplete data, and 1 was extracted unintentionally. This means that the degree of dislodgement was not assessed in 24 of 145 patients that received their allocated intervention (and 158 randomized) (Figure 1). One hundred twenty-one patients were included into the final analysis (Figure 1). Both groups were comparable with respect to age and gender. There were no significant differences between groups regarding puncture site, duration, access (midline versus paramedian), level (high, mid, and low thoracic) of catheterization, and type of surgery (Table 1).

### 3.1. Epidural Catheter Dislocation

**Figure 3:**
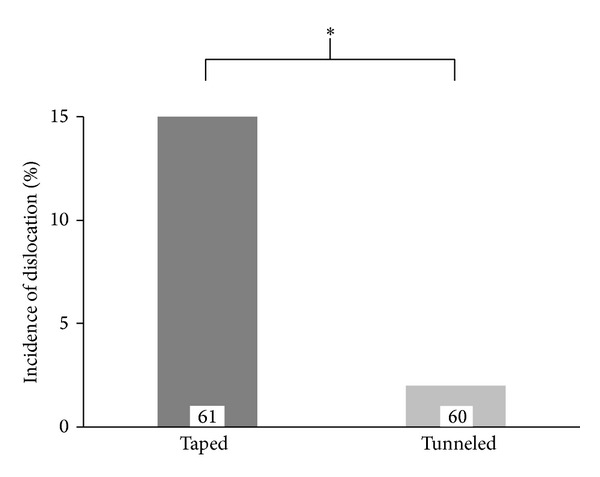
Incidence of catheter dislocation >20 mm. Data was available for *n* = 121 patients (61 taped/60 tunneled). Fisher's exact test was used to calculate statistical significance (^∗^ *P* < 0.01). Relative risk was 0.3389 [95% CI 0.1158–0.9920].

Tunneling and suture significantly decreased the incidence of catheter dislocation considered clinically relevant (>20 mm) from 9/61 (taped) to 1/60 (tunneled), respectively, (*P* < 0.01, Figure 3). Of all dislocations >20 mm, five epidurals of the taped group moved inwards; all other catheters moved outwards. Major displacement occurred mainly after day 2. No complications occurred by tunneling of the catheters. Particularly, we did not observe subcutaneous hematoma, bleeding, or occlusion of the catheter lumen by sutures placed too tight.

### 3.2. Quality of Postoperative Analgesia

**Figure 4:**
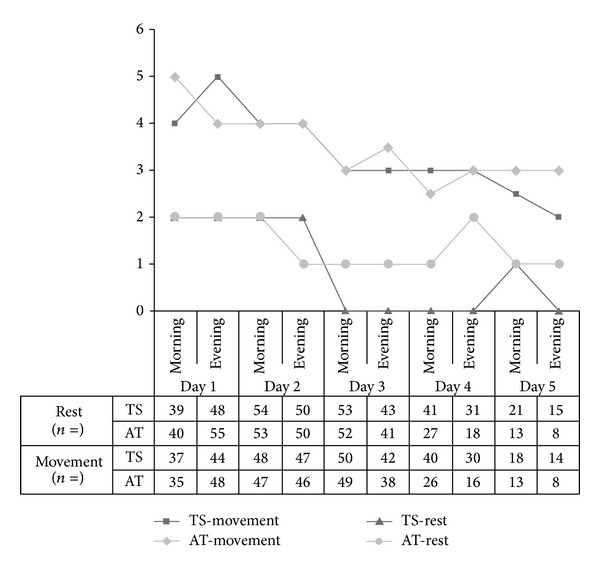
Comparison of analgesic quality by means of NRS (numeric rating scale). TS tunneled and sutured (“tunneled”); AT adhesive tape (“taped”). All data are presented as median. The total number of interviewed patients at different times is presented at the bottom of the graph. Not all patients were present at the time of the ward round.

Frequency of analgesic comedication as well as analgesic quality of both techniques was comparable between groups at rest as well as during movement over the course of time. When interviewed retrospectively, both groups showed no difference in satisfaction with the procedure, *P* = 0.26, (Figure 4).

### 3.3. Clinical Signs of Infection

All patients received systemic antibiotic medications as single shot surgical prophylaxis that was repeated once in patients with duration of surgery greater than 6 hours. No patient received antibiotics for EC-related infections. Overall, three patients presented with mild clinical signs of infection. One patient had a positive bacterial contaminated catheter (taped) and two patients had EC that were microbiologically sterile (tunneled). No patient showed signs of moderate or severe infection. Therefore, no extended diagnostics (blood cultures, MRI) were performed. There was no difference between the study groups.

### 3.4. Bacterial Contamination

**Figure 5:**
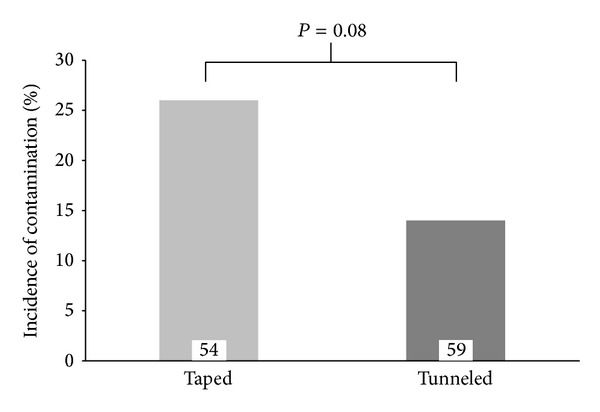
Overall incidence of bacterial contamination. 113 epidural catheter tips (59 tunneled, 54 taped) were available for microbiological screening. Fisher's exact test showed no statistical significance (*P* = 0.08).

Of 121 enrolled patients, 113 catheter tips (59 tunneled, 54 taped) were available for microbiological screening (Figure 5). Eight catheters were lost to followup. A total of 22 pathogens (8 tunneled, 14 taped EC) were detected. Tunneling and suture of EC tended to decrease bacterial contamination (*P* = 0.08). Coagulase-negative staphylococci (CoNS) were the predominant pathogens, exclusively found in the tunneled group and in the majority of the taped group, whereas* Staphylococcus aureus* and* Enterococcus faecalis* were isolated in two patients with catheters taped. Data with respect to contaminated EC are summarized in Table 2.

## 4. Discussion

Epidural catheter dislocation is a common phenomenon. Overall dislocation rate in this study was 37 percent (45/121), which is previously reported, though at the very high part of the range [4, 11–14]. The major achievement of our study is that we were able to demonstrate a more than 90 percent decrease of incidence of dislocation as compared to standard plain adhesive tape fixation. Furthermore, incidence of bacterial contamination tended to be decreased as well.

Premature catheter dislodgement bears relevant objective (economic) and subjective (patient) burden and may potentially lead to prolonged and more expensive inpatient stay [6]. In addition, unplanned catheter movement may be associated with rare, but clinically most relevant, complications such as spinal hematoma when occurring shortly after anticoagulant administration [14].

Epidural catheters in our institution are routinely inserted 3–5 cm into the epidural space in order to allow minor catheter movements without immediate loss of analgesic effect combined with lowest rates of catheter insertion-related problems (e.g., unilateral spread of anesthesia, neural root affection) [15, 16]. We routinely use multiorifice catheters with the most proximal orifice located 14 mm from catheter tip. If epidurals are inserted 30 mm a dislocation of 20 mm would consecutively lead more or less to procedural failure, as the proximal orifice would be out of the epidural space. Thus, we have chosen to adopt dislocation definitions introduced by Bougher et al. in 1996 [4], though a variety of other definitions exist [3, 11, 14, 17]. Dislocation rates in the tunneled group of our study were considerably lower in comparison to available literature [11, 13]. Only Tripathi and Pandey found comparable dislocation rates for tunneled EC of 3 percent [12]. There seems to be a tendency towards higher overall movement rates of thoracic in comparison to lumbar epidural catheters [4, 11]. As inward migration may lead to ascending levels of blockade or accidental dural perforation with consecutive spinal drug infusion, an even more stringent definition for clinically significant movement, usually 10 mm, has been suggested [3, 11, 13]. In our study, all patients had a thoracic epidural and received early daily physiotherapy; however, no case of secondary, accidental spinal drug infusion was reported. This study, to our knowledge, is the first to demonstrate the impact of using maximum effort of catheter fixation by a combination of techniques each described as independently reducing dislocation [3, 13].

Dislocation frequently occurs during treatment course (from day 2 on) and not directly after insertion [4, 11]. Table 2 shows that catheter dislodgment > 20 mm emerged around day four in the majority of cases. Clinically, day four of epidural treatment is distinguished by a nonsignificant accretion of pain intensity as expressed by NRS in the taped group only. We may speculate that reasons for late displacement may be postoperative recovery and increasing mobilization. Thus, tunneling and suture may be particularly beneficial if EC are planned to be used for more than a couple of days.

Bougher et al. did not report on any relation between catheter dislodgement and analgesia quality [4]. Bishton et al. in contrast found a 100 percent relation between catheter migration (≥25 mm) and failed epidural block [17]. Mourisse et al. observed that inward movement was accompanied by a higher level of sensory blockade but did not report on loss of analgesic quality [18]. We believe that routine use of analgetic comedication with NSAID (metamizole), paracetamol, and/or piritramide was sufficient enough to compensate the putative loss of late catheter function.

### 4.1. Clinical Signs of Infection

Tunneling and suture of epidurals may lead to local inflammatory reactions of the skin restricting a more prolonged use [12]. On the other hand, plain tape fixation theoretically allows less restricted in- and outward movement of catheters, thus potentially promoting infectious complications. Overall, three patients presented with signs of local infection (2.5%), which is comparable to earlier data from a German network [6]. As could be expected, no patient in our study suffered from moderate or severe infection and no catheter had to be removed in face of infectious complications. It was interesting to see a higher, though microbiologically unobtrusive, incidence with clinical signs of infection contamination in tunneled in comparison to taped epidurals, where* Staphylococcus aureus* was isolated. Factors presumably increasing the risk of infectious complications include age, gender, immunosuppression, duration of catheterization, and multiple punctures or puncture sites [6, 19]. In our study, these factors showed no statistical significant intergroup difference. It remains interesting that despite potential protective effects the rate of clinical signs of infection was twice as high for tunneled epidurals in this study, lending no support to the thesis that firm fixation is associated with less signs of infection. In contrast, the increased site inflammation can be readily explained by the increased number of skin punctures associated with tunneling and suturing. Given the extremely low incidence of severe, potentially fatal infectious complications like deep epidural infections, which varies from 0.007 percent (USA) to 0.025 percent (Sweden) [20, 21], it would be difficult to conduct a trial with sufficient power to detect any significant difference [10].

### 4.2. Bacterial Contamination

The total rate of pathogen findings in our study was 19% (22/113), with lower incidence for tunneled EC by trend (*P* = 0.08). Contamination rates found in literature vary from 4 percent to 53 percent [22, 23]. One possible explanation might be the use of propanol, an alcohol-based highly potent disinfectant, prior to catheter insertion. Positive microbiological cultures were defined as “bacterial contamination,” as accidental contamination during catheter removal could not be ruled out. Of note, blood cultures to confirm or exclude bacteremia were not taken. Yuan et al. suggested that bacterial migration along the epidural catheter track is the most common route of EC colonization [23]. But is there also relation between bacterial contamination and infectious complications?

The effect of subcutaneous tunneling for potentially preventing intravascular device-related infections has been shown [24]; however, its role in regional anesthesia is still a matter of scientific discussion. Bubeck et al. described a reduction of colonization of caudal catheters in children if tunneled, whereas Morin et al. could not observe any correlation between colonization and tunneling in regional anesthesia [25, 26]. At present there is no clear evidence for subcutaneous tunneling to prevent infections in regional anaesthesia [4]. Actual data from Germany stated tunneling rates in regional anesthesia of 21 percent unfortunately not discriminating between peripheral and central nerve blockades [6]. All of the pathogens identified in our study were Gram-positive and potentially capable of causing deep epidural infections (e.g., abscesses) [19]. It is difficult to assess the clinical impact of these findings, particularly, as contamination rarely leads to potentially life-threatening deep epidural infection [24] and the overall incidence of severe infectious complications is low [20, 21].

### 4.3. Limitations of the Study

As mentioned before, the degree of dislodgement was not assessed in 24 of 145 patients that received their allocated intervention, which may influence the true results. Specifically, premature removal, unintentional extraction, and change to PCIA are relevant outcomes because they may reflect dislodgement. The a priori power analysis was accomplished using the primary end-point of catheter dislocation considered to be clinically relevant. As infectious complications such as contamination are a rare event, this study is underpowered to detect statistical significant infectious complications of these secondary end-points of the study. Despite this lack of power with respect to infectious complications, a clear trend towards reduced bacterial contaminations using tunneling and suture was noted. Additionally, analgesia was achieved by epidural ropivacaine according to individual pain scores and not at a fixed per protocol rate. This might be considered another limit for the interpretation of analgesia between patient groups that cannot be resolved. Finally, both fixation techniques had been standardized and taught prior to inclusion of the first patient and photo illustrations of both techniques were available in each induction room. However, since the physician inserting the epidural catheter could not be blinded to the fixation technique we cannot completely exclude a “less cared” fixation contributing to the observed inferiority of taping epidural catheters.

## 5. Conclusions

Thorough tunneling and suture of thoracic epidural catheters significantly reduce incidence and extent of catheter dislocation and potentially that of bacterial contamination. Based on these results, we changed standards for patient care at our institution requiring catheter fixation by tunneling and suture in all patients receiving epidural catheters.

## Figures and Tables

**Table 1:** General data of study groups (tunneled versus taped).

	Tunneled	Taped	
Age [years]	57	58	*P* = 0.34
Gender [male : female]	35 : 25	35 : 26	*P* = 0.61
Duration of catheterization [hours]	109 (±46)	97 (±37)	<*P* = 0.06
Access of puncture (*n*=)			*P* = 0.2
Midline	38	44	
Paramedian	22	17	
Level of puncture (*n*=)			*P* = 0.2
High thoracic (T_3/4_–T_6/7_)	7	10	
Mid thoracic (T_7/8_–T_8/9_)	40	41	
Low thoracic (T_9/10_–T_11/12_)	13	10	
Type of surgery (*n*=)			*P* = 0.31
Thoracic	7	9	
Abdominal	44	37	
Urological	8	15	
Combined	1	0	

General data revealed no intergroup difference. Student's *t*-test, Fisher's exact test, and Mann-Whitney *U* test were used for statistical analysis.

**Table 2:** Data concerning contaminated epidural catheters.

	Age	Sex	Site	Level	Duration (h)	Dislocation	Pathogen	Comorbidities				Perioperative antibiotics	Punctures	Signs of infection
								MG	DM	CS	CT			
Group 1 (tunneled)	47	M	Paramedian	Mid	147		CoNS∗	Yes	No	No	Yes	CPZ^¶^ (2 g)	1 x	Ø
	61	M	Midline	High	294		CoNS∗	Yes	No	No	No	CPZ^¶^ (2 g) + METRO∗∗ (0.5 g)	2 x (os)	Ø
	62	F	Midline	Mid	99		CoNS∗	Yes	No	No	No	CPZ^¶^ (2 g) + METRO∗∗ (0.5 g)	1 x	Ø
	76	F	Midline	Mid	100		CoNS∗	Yes	No	No	No	CPZ^¶^ (2 g) + METRO∗∗ (0.5 g)	1 x	Ø
	79	F	Midline	Mid	74	10 mm out	CoNS∗	Yes	No	No	Yes	CPZ^¶^ (2 g) + METRO∗∗ (0.5 g)	2 x (os)	Ø
	44	M	Midline	Low	48		CoNS∗	No	No	No	No	CPZ^¶^ (2 g)	1 x	Ø
	36	M	Midline	Low	124		CoNS∗	Yes	No	No	No	CFTX^††^ (2 g)	1 x	Ø
	61	M	Midline	High	101		STAEPI^†^	Yes	No	No	No	CPZ^¶^ (2 g)	1 x	Ø

Group 2 (taped)	55	M	Midline	Mid	76		CoNS∗	Yes	No	No	No	CPZ^¶^ (2 g)	1 x	Ø
	68	M	Paramedian	Mid	124		CoNS∗	Yes	No	No	No	CPZ^¶^ (2 g) + METRO∗∗ (0.5 g)	1 x	Ø
	78	F	Midline	Mid	93	30 mm in	CoNS∗	Yes	Yes	No	No	CPZ^¶^ (2 g) + METRO∗∗ (0.5 g)	2 x	Ø
	66	M	Midline	Mid	96		CoNS∗	Yes	No	No	No	CPZ^¶^ (2 g) + METRO∗∗ (0.5 g)	1 x	Ø
	53	M	Midline	Mid	194		CoNS∗	Yes	No	No	No	CPZ^¶^ (2 g) + METRO∗∗ (0.5 g)	1 x	Ø
	75	F	Midline	Mid	76	10 mm out	CoNS∗	Yes	Yes	No	No	CPZ^¶^ (2 g)	1 x	Ø
	71	M	Midline	Low	70		CoNS∗	Yes	No	No	No	CPZ^¶^ (2 g) + METRO∗∗ (0.5 g)	1 x	Ø
	53	M	Midline	High	99	20 mm out	STAAUR^‡^	Yes	No	No	Yes	CPZ^¶^ (2 g) + METRO∗∗ (0.5 g)	1 x	Redness, swelling
	76	M	Midline	Mid	190		CoNS∗	Yes	Yes	No	No	CPZ^¶^ (2 g) + METRO∗∗ (0.5 g)	1 x	Ø
	83	F	Paramedian	Low	148		CoNS∗	Yes	No	No	No	CPZ^¶^ (2 g) + METRO∗∗ (0.5 g)	1 x	Ø
	66	F	Midline	Low	100		CoNS∗	Yes	No	Yes	No	CFTX^††^ (2 g)	1 x	Ø
	61	M	Paramedian	High	96	90 mm out	CoNS∗ + ENCFIS^§^	Yes	No	No	No	CPZ^¶^ (2 g)	1 x	Ø
	37	M	Midline	Mid	167		CoNS∗	No	No	No	No	CPZ^¶^ (2 g) + METRO∗∗ (0.5 g)	1 x	Ø
	53	M	Midline	Mid	95	20 mm in	CoNS∗	No	No	Yes	No	CPZ^¶^ (2 g) + METRO∗∗ (0.5 g)	1 x	Ø

*P*=	**0.2**	**0.5**	**0.9**	**0.4**	**0.4**									

MG: malignancy; DM: diabetes mellitus; CS: corticosteroids; CT: chemotherapy; ∗CoNS: coagulase-neg. staphylococci; ^†^STAEPI: *Staphylococcus epidermidis*; ^‡^STAAUR: *Staphylococcus aureus*; ^§^ENCFIS: *Enterococcus faecalis*; ^¶^CPZ: cephazoline; ∗∗METRO: metronidazole; ^††^CFTX: ceftriaxone; antibiotics were administered exclusively in the perioperative period. Note that none of the contaminated epidural catheters in the tunneled and only one epidural catheter *n* the taped group showed clinical signs of infection. Student's *t*-test, Fisher's exact test, and Mann-Whitney *U* test were used for statistical testing. Of note, the patient of the taped group with a 90 mm catheter dislodgment showed no signs of insufficient analgesia during rest; however, his visual analogue scale was 5-6 on movement for the first three days.
